# The relationship between social support and physical activity among college students: the chain mediation of peer relationships and exercise self-efficacy

**DOI:** 10.3389/fpsyg.2026.1873443

**Published:** 2026-10-21

**Authors:** Jiawei Chen, Wu Zhou

**Affiliations:** School of Physical Education, Hangzhou Normal University, Hangzhou, China

**Keywords:** chained mediation, exercise self-efficacy, peer relationships, physical activity, social support

## Abstract

**Objective:**

Drawing on social cognitive theory, this study examines the relationship between social support and physical activity among university students, as well as the independent and chained mediating effects of peer relationships and exercise self-efficacy, with a view to providing a theoretical basis and evidence-based support for physical education and health promotion initiatives in higher education institutions.

**Methods:**

Using a stratified sampling method, a survey was conducted among 749 university students, employing the Social Support Scale, the Physical Activity Scale, the Peer Relationship Scale and the Exercise Self-Efficacy Scale. After controlling for gender and academic year, analyses were performed using the heterogeneous-homogeneous correlation ratio, multiple linear regression and Model 6 of the Hayes PROCESS macro; mediating effects were tested via 5,000 Bootstrap repetitions.

**Results:**

(1) Social support and peer relationships demonstrated good discriminant validity, and social support was found to be a significant positive predictor of physical activity among university students (*β* = 0.301, *p* < 0.01). After controlling for mediating variables, its direct effect remained significant (*β* = 0.088, *p* < 0.05). (2) Peer relationships and exercise self-efficacy both exert independent mediating effects between social support and physical activity; furthermore, the two form a significant chained mediation effect. (3) The effect sizes of the three indirect pathways were 0.194, 0.030 and 0.023, respectively, accounting for 55.37%, 8.64 and 6.61% of the total effect; the 95% confidence intervals for all three did not include zero.

**Conclusion:**

Social support is not only directly associated with university students’ participation in physical activity, but may also exert an indirect influence through independent and chained mediation pathways involving peer relationships and exercise self-efficacy. Among these, peer relationships constitute the core pathway through which the effects of social support are transmitted. This study reveals the psychosocial mechanism of ‘interpersonal interaction—cognitive restructuring—behavioral practice’ linking social support to university students’ physical exercise behavior, providing important insights for refining physical education intervention strategies in higher education institutions.

## 1 Introduction

Among Chinese university students, both overall physical activity and participation in moderate-to-vigorous physical activity during physical education classes have been associated with better mental health (Xu et al., 2025). Physical activity refers to any bodily movement produced by skeletal muscles that results in energy expenditure; regular physical activity helps to reduce the risk of non-communicable diseases and improve mental health. The WHO’s 2020 guidelines recommend that adults engage in 150–300 min of moderate-intensity aerobic activity, or 75–150 min of vigorous-intensity aerobic activity, per week, and also undertake muscle-strengthening activities (Bull et al., 2020). However, a meta-analysis of 507 population-based surveys revealed that the global prevalence of insufficient physical activity among adults reached 31.3% in 2022, a significant increase from 23.4% in 2000 (Strain et al., 2024). University students are at a critical stage of transition from adolescence to early adulthood, during which their learning styles, daily routines and social relationships are undergoing a process of restructuring. Insufficient physical activity not only affects their current physical health but may also impact the long-term development of healthy behavioral habits.

In recent years, research into physical activity among university students has gradually shifted from identifying single factors to a combined explanation involving the social environment and individual cognition. A 2024 meta-analysis incorporating 19 studies revealed that social support is significantly positively correlated with physical activity among university students, and that the association between support from friends and physical activity is stronger than that with support from family (Wang et al., 2024); a three-wave study of Chinese university students further found that changes in peer support can predict the developmental trajectories of total physical activity, duration and frequency (Cao et al., 2023). At the same time, social support may influence university students’ participation in physical exercise by enhancing their sense of self-efficacy; this mediating pathway has been supported by recent empirical studies and meta-analyses (Lin et al., 2024; Niu et al., 2025; Zhang et al., 2022). It is therefore evident that social support is not merely an external factor; its effects may also be translated into physical activity through university students’ daily peer interactions and beliefs about their own capabilities. A review of the existing literature reveals that, while a relatively robust evidence base has been established, there remains scope for further examination of the sequential relationships between variables. On the one hand, existing studies involving university students have predominantly examined a single mediating pathway: ‘social support → exercise self-efficacy → physical activity’; on the other hand, recent chained models have either placed peer relationships as the antecedent and physical activity intention as the outcome variable (Zhou et al., 2025), or tested the sequence ‘social support → self-efficacy → peer relationships’ in relation to general health behaviors among adolescents (Lu et al., 2024). While the aforementioned studies have confirmed the close link between social support, peer relationships and self-efficacy, they have not yet fully addressed the question of whether, in a university setting, broader social support can first be transformed into high-quality peer relationships and subsequently promote actual physical activity by enhancing self-efficacy.

Social cognitive theory emphasizes that support from the external environment must undergo cognitive processing and behavioral experience by the individual before it can be transformed into stable behavior; self-efficacy is a key cognitive mechanism in this transformation process (Bandura, 2004; Beauchamp et al., 2019). For university students, social support from family, teachers and peers can provide information on exercise, opportunities for participation and emotional encouragement, while positive peer relationships enable this support to manifest concretely in everyday interactions through joint exercise, role modeling and positive feedback. Through joint participation and ongoing interaction with peers, university students may continuously accumulate physical activity experience and gain confirmation of their abilities, thereby strengthening their confidence in overcoming fatigue, time constraints and environmental limitations, which in turn leads to a higher sense of self-efficacy regarding exercise and helps them maintain physical activity. Consequently, the impact of social support on physical activity may not be a simple direct effect, but rather a continuous process in which supportive resources are realized through peer interactions and are further transformed into beliefs about one’s own capabilities. However, whether this continuous mechanism can explain university students’ actual levels of physical activity remains to be further tested. Accordingly, this study takes university students as its research subjects and constructs a chained mediation model comprising ‘social support—peer relationships—exercise self-efficacy—physical activity’. It examines the relationship between social support and physical activity, and tests the independent and chained mediating effects of peer relationships and exercise self-efficacy, with a view to deepening our understanding of the mechanisms underlying physical activity among university students and providing empirical evidence to assist higher education institutions in enhancing students’ levels of physical activity.

## 2 Literature review and hypothesis formulation

### 2.1 The relationship between social support and physical activity

Social support refers to the system of material, informational, and emotional assistance and support that individuals receive from others. For college students in early adulthood, social support from multiple sources is vital for coping with external challenges and maintaining physical and mental well-being (Gottlieb and Bergen, 2010). Adequate social support can provide college students with necessary exercise resources, create a positive exercise environment, and strengthen their motivation to exercise, thereby effectively increasing their physical activity levels. Research indicates that social support is a key environmental factor influencing an individual’s physical activity (Ståhl et al., 2001). For example, encouragement and companionship from family members can significantly increase adolescents’ exercise frequency; meanwhile, shared participation, mutual encouragement, and emotional support among friends and classmates can satisfy college students’ need for a sense of belonging, making physical exercise a more attractive social activity and thereby increasing their participation. Furthermore, according to the main effects model of social support, social support plays a positive role in promoting an individual’s physical and mental health as well as behavioral adaptation; that is, high levels of social support can provide individuals with a stable sense of belonging, self-worth, and security, thereby directly motivating them to maintain healthy behavioral patterns (Cohen and Wills, 1985). Based on this, this study proposes *Hypothesis H1*: Social support can positively predict physical activity among college students.

### 2.2 The relationship between social support, peer relationships, and physical activity

Peer relationships refer to interpersonal relationships established and developed through interaction between individuals of similar age, status and cognitive level. According to social cognitive theory, an individual’s behavior is the result of the interaction between environmental and personal factors (Bandura, 2001). On the one hand, peer relationships serve as a key vehicle through which social support influences individual behavior. The encouragement and support that university students receive from teachers and family members can enhance their sense of social belonging and social confidence, thereby helping them to establish broader and more harmonious peer relationships with their peers (Wu and Dong, 2024). The establishment and development of interpersonal relationships is a process that gradually deepens through the exchange of information, emotions and resources. During this process, the broad support individuals receive from their social networks can reduce their sense of risk and psychological burden associated with self-disclosure, enabling them to engage more proactively and confidently in peer interactions, thereby accelerating the deepening of interpersonal relationships. Previous research has also demonstrated that the level of social support perceived by university students is significantly and positively correlated with the quality of their peer relationships; individuals who feel more supported tend to have more harmonious peer relationships. It is therefore evident that social support is a significant positive predictor of peer relationships (Boling et al., 2011).

On the other hand, an individual’s behavior is constrained and driven by the psychological dynamics of the group to which they belong. In the context of physical exercise, peers collectively form a dynamic group within which specific goals, norms and attractions emerge. When an individual is part of a group of peers who are passionate about sport and actively participate, the group establishes a powerful behavioral norm that implicitly regards regular physical activity as an accepted and encouraged group behavior. Through psychological mechanisms such as peer imitation, conformity and comparison, this norm generates motivation in individuals to increase their physical activity (Harden et al., 2015). Smith et al. (2015) further pointed out that positive peer relationships can enhance an individual’s motivation to engage in physical exercise. At the same time, the collaborative contexts created by physical education and health courses in higher education institutions mean that the positive peer relationships formed during these activities become a key factor driving students to engage more proactively in physical exercise. It is therefore evident that peer relationships can serve as a positive predictor of physical activity (Smith et al., 2015). On this basis, this study proposes Hypothesis H2: peer relationships mediate the relationship between social support and physical activity.

### 2.3 The relationship between social support, self-efficacy, and physical activity

Exercise self-efficacy refers to an individual’s confidence in their ability to perform and complete physical exercise tasks and overcome related obstacles (Neace et al., 2022). Bandura believes that an individual’s sense of self-efficacy is primarily shaped by successful experiences, vicarious experiences, verbal persuasion, and physiological and emotional states (Su et al., 2026). Furthermore, building upon the emphasis on environmental factors in social cognitive theory, classical social support theory further posits that social support provides individuals with emotional comfort, informational assistance, and material support (House, 1981). When facing stress and adversity, support from family, friends, peers, and professionals serves as a solid foundation, enhancing an individual’s confidence and ability to cope with challenges. Research further indicates that social support, derived from the encouragement and support of others, can significantly enhance an individual’s self-efficacy (Zhang et al., 2022). It can therefore be inferred that, among college students, social support may positively predict an individual’s exercise self-efficacy.

Furthermore, social cognitive theory suggests that self-efficacy influences an individual’s behavior primarily through cognitive, motivational, emotional and decision-making processes. Individuals with high exercise self-efficacy set more challenging exercise goals for themselves and invest more effort in achieving them; conversely, those with low self-efficacy may give up trying altogether because they anticipate failing. Research also indicates that high levels of exercise self-efficacy are closely associated with high exercise frequency and active participation in exercise (Han et al., 2022). Therefore, among college students, exercise self-efficacy may positively predict physical activity. Based on this, this study proposes Hypothesis H3: Exercise self-efficacy mediates the relationship between social support and physical activity.

### 2.4 The chain mediation hypothesis of peer relationships and exercise self-efficacy

According to the findings of the aforementioned review, social support, peer relationships and self-efficacy regarding exercise are closely associated with physical activity. In particular, social support may be indirectly associated with physical activity via peer relationships and exercise self-efficacy, respectively. However, is there an intrinsic, sequential relationship between these two mediating variables—peer relationships and exercise self-efficacy? Some studies have shown that there is a close association between peer relationships and self-efficacy. Existing research has found a significant positive correlation between peer support and self-efficacy; encouragement, companionship and behavioral feedback from peers may serve as important sources of social information for individuals in forming positive assessments of their own capabilities (Zou et al., 2023). Positive peer relationships provide university students with valuable resources for observational learning, joint training and mutual encouragement, thereby laying the foundation for individuals to enhance their self-efficacy. Other studies have also found that, in physical activities, positive peer relationships significantly enhance individuals’ confidence in sport and their perception of their abilities through mechanisms such as providing alternative experiences, verbal persuasion and the sharing of experiences of success and failure (Ren et al., 2020). When university students receive guidance, encouragement and collaborative support from their peers during physical exercise, they tend to assess their ability to complete exercise tasks more positively, thereby enhancing their self-efficacy in exercise. From a triadic interaction perspective based on social cognitive theory, there are internal interrelationships between environmental, individual and behavioral factors. In other words, broad social support facilitates the formation of positive peer relationships, and these peer relationships—established through shared physical activities—further reinforce an individual’s exercise self-efficacy by providing abundant information regarding efficacy, ultimately leading to more active participation in physical activity. Based on this, the present study proposes Hypothesis 4: peer relationships and exercise self-efficacy play a chained mediating role in the relationship between social support and physical activity (Figure 1).

**Figure 1:**
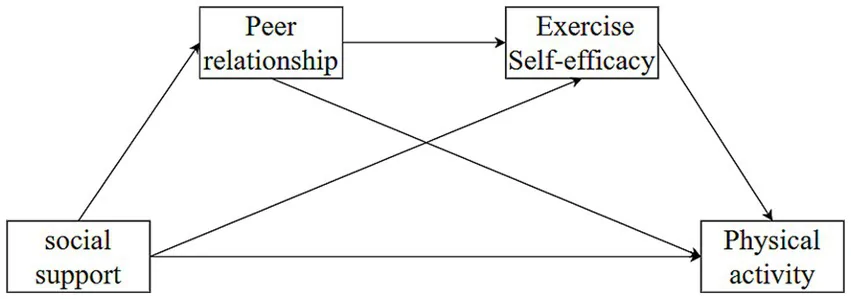
Assumption model diagram.

## 3 Research design and methods

### 3.1 Design

This study employs a cross-sectional design, with social support as the independent variable, physical activity among university students as the dependent variable, and peer relationships and exercise self-efficacy as mediating variables. Prior to the commencement of the study, a prior power analysis was conducted using R 4.6.1 and the pwr package. In accordance with the structure of the chained mediation model employed in this study, a fixed-effects multiple regression model was utilized, with the maximum number of predictors set at 3. Under the conditions of an effect size of Cohen’s *f*^2^ = 0.15, a significance level of *α* = 0.05, and a statistical power of 0.90, the required minimum sample size was calculated to be 99 participants. This study ultimately included a valid sample of 749 participants, which far exceeded the minimum sample size requirement, indicating that the study possessed sufficient statistical power. Subsequently, using stratified sampling, Province A was divided into five regions—Eastern, Western, Northern, Southern, and Central—with one higher education institution randomly selected from each region. Prior to the survey, online training was provided to student counselors at each institution, requiring them to strictly ensure that the survey was conducted on a voluntary basis and to inform students that the information provided would be used solely for academic purposes. Following their consent, the questionnaire was distributed via the Questionnaire Star platform from 1 November 2025 to 20 December 2025. A total of 996 questionnaires were collected; 249 were excluded on the grounds of ‘completion time under 3 min’, ‘incomplete responses’ and ‘patterned responses’, 249 invalid questionnaires were excluded, leaving 749 valid responses, with a valid response rate of 75.2%. Among the respondents, 301 were male, accounting for 40.2%, and 448 were female, accounting for 59.8%.

### 3.2 Methods

#### 3.2.1 Social Support Scale

This study employed the Social Support Scale revised by Ye and Dai (2008), which comprises three dimensions—subjective support, objective support, and utilization of support—totaling 17 items, to assess individuals’ status within their social support systems. In this study, the Cronbach’s alpha coefficient for the scale was 0.948. Furthermore, confirmatory factor analysis revealed the following indices: *χ*^2^/df = 1.835, GFI = 0.975, CFI = 0.987, IFI = 0.989, TLI = 0.987, and RMSEA = 0.034, indicating that the scale demonstrates good reliability and validity.

#### 3.2.2 Physical Activity Scale

This study utilized the Physical Activity Rating Scale-3 (PARS-3), revised by Liang Deqing et al., to assess the level of physical exercise among university students. The scale comprises three items: exercise intensity, duration of a single exercise session, and frequency of exercise, and employs a five-point Likert scale (1 = never, 2 = rarely, 3 = sometimes, 4 = often, 5 = always) (Dq, 1994). An individual’s level of physical activity is measured by the product of the scores across three components. According to the classification criteria of the original scale, a total score of ≤19 indicates a low level of physical activity, 20–42 indicates a moderate level, and ≥43 indicates a high level. This scoring method takes into account exercise intensity, duration and frequency, thereby avoiding the assessment of physical activity levels based on a single indicator alone. The structure and standard scoring method of the PARS-3 have also been consistently applied in studies of Chinese university students. A higher score on the scale indicates a higher level of physical activity. In this study, the Cronbach’s α coefficient for this scale was 0.705.

#### 3.2.3 Peer Attachment Scale

This study employed the Peer Attachment Scale developed by Armsden and Greenberg (1987), which consists of 25 items designed to measure the attachment relationship between individuals and their peers and can systematically reflect the characteristics of peer attachment. The scoring method uses a 5-point Likert scale, ranging from “strongly disagree” to “strongly agree,” with scores assigned from 1 to 5; this scale is widely used and can reliably and accurately measure the attachment relationship between an individual and a peer. In this study, the Cronbach’s alpha coefficient for this scale was 0.906. Confirmatory factor analysis further revealed the following indices: *χ*^2^/df = 1.660, GFI = 0.968, CFI = 0.987, IFI = 0.987, TLI = 0.986, and RMSEA = 0.030, indicating that the scale possesses good reliability and validity.

#### 3.2.4 Self-Efficacy Scale

The “Exercise Self-Efficacy” scale, developed by Resnick and Jenkins in 2000, was used. This scale consists of 9 items and employs an 11-point Likert scale. It is primarily designed to assess individuals’ perceptions of their exercise capabilities in various situations, such as during inclement weather or when fatigued, and effectively measures individuals’ perceived exercise capabilities under different conditions (Resnick and Jenkins, 2000). In this study, the Cronbach’s alpha coefficient for this scale was 0.944. The results of confirmatory factor analysis showed: *χ*^2^/df = 1.940, GFI = 0.991, CFI = 0.995, IFI = 0.995, TLI = 0.995, and RMSEA = 0.035, indicating that the scale has good reliability and validity.

#### 3.2.5 Statistical methods and data processing

This study used SPSS 27.0 to conduct normality tests, descriptive statistics, correlation analysis, *t*-tests, and analysis of variance (ANOVA). The Hayes Process was used to test for mediating effects, with 5,000 bootstrap samples. AMOS 26.0 was used to perform confirmatory factor analysis (CFA). The normality of the variables was assessed using West’s skewness and kurtosis criteria. Quantitative data are described as mean ± standard deviation (*M* ± SD).

### 3.3 Common method bias test

The purpose of common method bias detection is to avoid the artificial amplification or masking of correlations between variables resulting from a single data source, thereby enhancing the reliability and rigor of the study. Following the guidelines of Podsakoff et al. (2003) this study employed Harman’s one-factor test to assess common method bias (Harman, 1976). Through unrotated principal component analysis, eight factors with eigenvalues greater than 1 were extracted. Among these, the first factor explained 35.673% of the variance, which did not reach the critical threshold of 40%, indicating that this study does not exhibit serious common method bias.

## 4 Research findings and analysis

### 4.1 Descriptive statistics and correlation analysis

This study used SPSS 27.0 to perform descriptive statistics and correlation analysis on the sample. Based on the scoring method, social support, physical activity, and peer relationships were all set with a median of 3, while exercise self-efficacy was set with a median of 6. The results indicate that among college students, social support and peer relationships are relatively strong, while physical activity and exercise self-efficacy still need improvement.

The results of the correlation analysis indicate that social support is significantly positively correlated with physical activity (*r* = 0.341), peer relationships (*r* = 0.621), and exercise self-efficacy (*r* = 0.335, *p* < 0.01); physical activity was significantly positively correlated with peer relationships (*r* = 0.403) and exercise self-efficacy (*r* = 0.366, *p* < 0.01); peer relationships were also significantly positively correlated with exercise self-efficacy (*r* = 0.33, *p* < 0.01) (Table 1).

**Table 1:** Correlation coefficient between variables.

Variable	Social support	Physical activity	Partnership	Building Self-Efficacy
Social support	1			
Physical activity	0.341**	1		
Partnership	0.621**	0.403**	1	
Building Self-Efficacy	0.335**	0.366**	0.334**	1
*M*	4.031	2.882	3.676	6.401
SD	0.785	0.917	0.550	2.325

“**” indicates *p* < 0.01.

### 4.2 Analysis of demographic control variables

#### 4.2.1 Independent samples *t*-test for gender

According to West’s criteria for testing the normality of a sample distribution, the hypothesis that the sample is normally distributed is accepted when the absolute values of skewness and kurtosis are less than 3 and 10, respectively. The results of this study show that the absolute values of skewness (−1.440 to 0.537) and kurtosis (−1.305 to 1.685) for each item are all below the criteria proposed by West; therefore, subsequent t-tests and analysis of variance (ANOVA) may be conducted.

The gender difference test revealed that male students’ social support scores (4.182 ± 0.725) were significantly higher than those of female students (3.929 ± 0.808), *t* = 4.379, *p* < 0.001, Cohen’s *d* = 0.326; Male students’ peer relationship scores (3.755 ± 0.512) were also significantly higher than those of female students (3.623 ± 0.569), *t* = 3.240, *p* = 0.001, Cohen’s *d* = 0.241. Boys’ exercise self-efficacy (7.401 ± 2.251) was significantly higher than that of girls (5.729 ± 2.126), Welch’s *t* = 10.300, *p* < 0.001, Cohen’s *d* = 0.768; Male students’ physical activity scores (3.272 ± 0.872) were also significantly higher than those of female students (2.620 ± 0.851), *t* = 10.188, *p* < 0.001, Cohen’s *d* = 0.759. Overall, the gender differences in social support and peer relationships were small, while those in exercise self-efficacy and physical activity were moderate and approached large effects (Cohen, 1988).

Tests for differences across educational stages revealed that students at different stages differed in terms of social support, *F* = 9.498, *p* < 0.001, *η*^2^ = 0.037; peer relationships, *F* = 3.473, *p* = 0.016, *η*^2^ = 0.014; and exercise self-efficacy, *F* = 7.276, *p* < 0.001, *η*^2^ = 0.028; although the effect sizes were all small. There were no significant differences in physical activity among students across different educational stages, *F* = 1.955, *p* = 0.119, *η*^2^ = 0.008. Given that gender was significantly associated with the main study variables and that there were slight differences across educational stages for some variables, both the subsequent hierarchical regression and chained mediation analyses treated gender and educational stage as control variables.

### 4.3 Analysis of mediating effects

#### 4.3.1 Hypothesis testing for regression models

Prior to conducting hierarchical regression and chained mediation analyses, this study tested for multicollinearity, linearity and homoscedasticity. The variance inflation factors (VIFs) for the predictor variables in each model ranged from 1.029 to 1.715, with tolerance values ranging from 0.583 to 0.971, indicating that there was no severe multicollinearity. The Ramsey RESET test revealed no significant deviations from linearity in the peer relationships equation or the physical activity equation (*p* = 0.400 and *p* = 0.202, respectively), but there were some indications of non-linearity in the exercise self-efficacy equation (*p* = 0.011). The Breusch–Pagan test revealed no significant heteroscedasticity in the exercise self-efficacy equation (*p* = 0.164), while heteroscedasticity was observed in the peer relationships equation and the physical activity equation (*p* = 0.003 and *p* < 0.001, respectively). Consequently, this study did not report HC3 heteroscedasticity-robust standard errors in the hierarchical regression analysis; instead, 95% confidence intervals for direct and indirect effects were estimated using 5,000 Bootstrap repetitions to mitigate the impact of heteroscedasticity and the non-normal distribution of indirect effects on statistical inference.

#### 4.3.2 Hierarchical regression analysis

To examine the incremental explanatory power of each variable on physical activity, this study employed hierarchical multiple linear regression and constructed four nested models in accordance with theoretical sequence. Model 1 included gender and school stage as covariates; Model 2 added social support; Model 3 further included peer relationships; and Model 4 added exercise self-efficacy (Table 2).

**Table 2:** Regression results of chain mediation effect (*n* = 749).

Variable	M1			M2			M3			M4		
	*b*	*SE*	*β*	*b*	*SE*	*β*	*b*	*SE*	*β*	*b*	*SE*	*β*
Gender	0.653	0.064	0.350**	0.561	0.062	0.300**	0.550	0.060	0.295**	0.457	0.063	0.245**
Grade	−0.010	0.059	−0.007	0.062	0.054	0.042	0.056	0.050	0.038	0.064	0.049	0.044
Social support				0.351	0.044	0.301**	0.133	0.051	0.114**	0.103	0.051	0.088*
Peer relationship							0.500	0.070	0.300**	0.447	0.069	0.268**
Exercise relationship										0.064	0.015	0.163**
*R* ^2^	0.122			0.208			0.263			0.284		

Gender is coded as 0 for female and 1 for male; year of study is coded as 1 for first year, 2 for second year, 3 for third year and 4 for fourth year; *p < 0.05, **p < 0.01. The year-of-study coefficient represents the average linear change for each increase in year of study. b denotes the unstandardized regression coefficient, β denotes the standardized regression coefficient, and SE denotes the HC3 heteroscedasticity-robust standard error.

In Model 1, gender had a significant positive effect on physical activity (*β* = 0.350, *p* < 0.01), indicating that boys scored higher on physical activity than girls; the linear effect of school stage was not significant (*β* = −0.007, *p* = 0.838). In Model 2, after including social support, gender continued to exert a significant positive influence (*β* = 0.300, *p* < 0.01), while the effect of educational stage remained non-significant (*β* = 0.042, *p* = 0.203); furthermore, higher levels of social support were associated with higher levels of physical activity (*β* = 0.301, *p* < 0.01).

In Model 3, peer relationships were included, and gender remained significant (*β* = 0.295, *p* < 0.01), while the school stage remained non-significant (*β* = 0.038, *p* = 0.231); both social support (*β* = 0.114, *p* < 0.01) and peer relationships (*β* = 0.300, *p* < 0.01) exerted significant positive effects. In Model 4, exercise self-efficacy was added, and the results followed the same pattern: gender had a significant positive effect (*β* = 0.245, *p* < 0.01), while the linear effect of educational stage remained non-significant (*β* = 0.044, *p* = 0.165); social support (β = 0.088, *p* < 0.05), peer relationships (*β* = 0.268, *p* < 0.01) and exercise self-efficacy (*β* = 0.163, *p* < 0.01) all showed that higher scores were associated with higher levels of physical activity.

#### 4.3.3 Testing for chain mediation effects

With social support as the independent variable and physical activity as the dependent variable, and peer relationships and exercise self-efficacy as chained mediating variables, the analysis was conducted using Model 6 in the Hayes PROCESS macro, with 5,000 Bootstrap repetitions, while controlling for gender and school stage. The results showed that the total effect of social support on physical activity was *B* = 0.351 (*β* = 0.301, Boot SE = 0.043, 95% CI [0.265, 0.436]); after including the two mediating variables, the direct effect was *B* = 0.103 (*β* = 0.088, Boot SE = 0.050, 95% CI [0.004, 0.204]). The total indirect effect was *B* = 0.248 (*β* = 0.212, Boot SE = 0.034, 95% CI [0.182, 0.317]), accounting for 70.62% of the total effect. The confidence intervals for all three specific indirect pathways exclude 0: the indirect effect of social support on physical activity via peer relationships was *B* = 0.194 (*β* = 0.166, Boot SE = 0.032, 95% CI [0.132, 0.257]), accounting for 55.37% of the total effect; the indirect effect of social support on physical activity via exercise self-efficacy was *B* = 0.030 (*β* = 0.026, Boot SE = 0.011, 95% CI [0.011, 0.055]), accounting for 8.64%; The chained indirect effect of social support on physical activity via peer relationships and exercise self-efficacy was *B* = 0.023 (*β* = 0.020, Boot SE = 0.007, 95% CI [0.011, 0.038]), accounting for 6.61%. Both the direct effects and the total indirect effects were significant, indicating that peer relationships and exercise self-efficacy partially mediate the relationship between social support and physical activity. Fully standardized effect β was used to characterize the effect size; specific results are presented in Figure 2 and Table 3.

**Figure 2:**
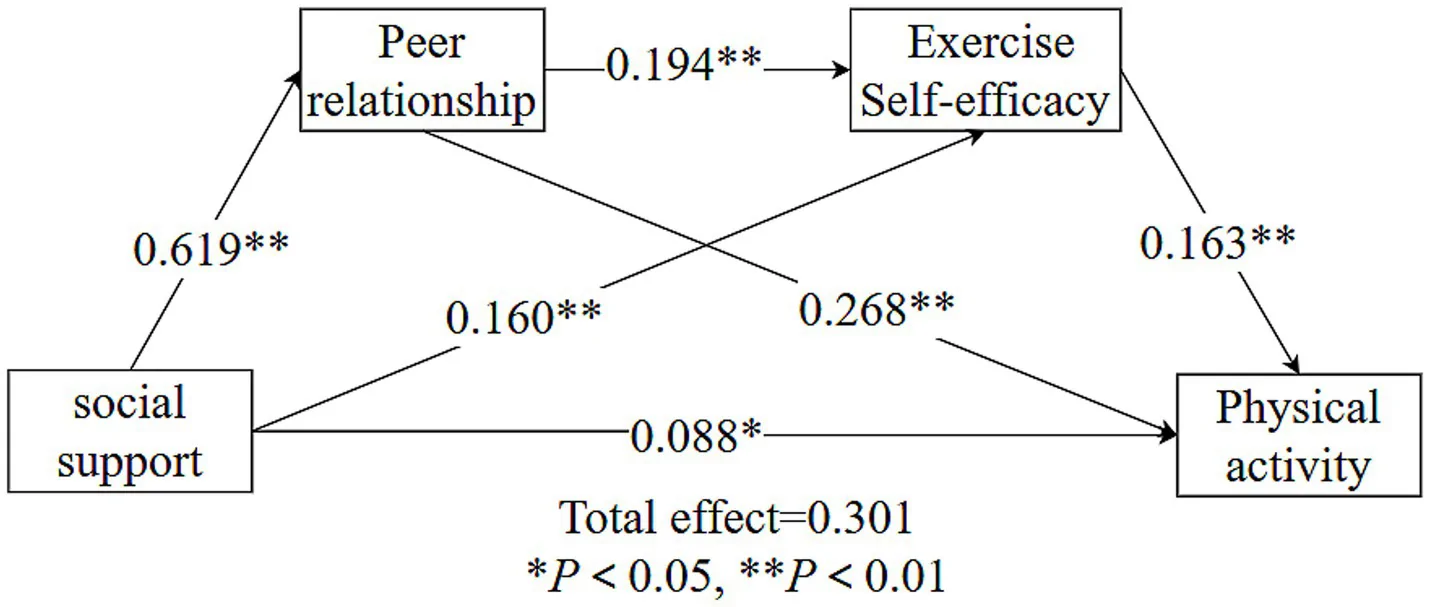
Diagram of the chain brokerage model.

**Table 3:** Direct and indirect effects after controlling for gender and the linear effect of academic stage (*n* = 749).

Effects and pathways	*B*	*β*	Bootstrapped standard error	Bootstrap 95% CI		Relative effect size
				LLCI	ULCI	
Total effect	0.351	0.301	0.043	0.265	0.436	100.00%
Direct effect	0.103	0.088	0.050	0.004	0.204	29.38%
Total indirect effect	0.248	0.212	0.034	0.182	0.317	70.62%
Path 1: Social support → Peer relationships → Physical activity	0.194	0.166	0.032	0.132	0.257	55.37%
Path 2: Social support → Exercise self-efficacy → Physical activity	0.030	0.026	0.011	0.011	0.055	8.64%
Path 3: Social support → Peer relationships → Exercise self-efficacy → Physical activity	0.023	0.020	0.007	0.011	0.038	6.61%

B represents the unstandardized effect; β represents the fully standardized effect and serves as a measure of the indirect effect; Boot standard errors and 95% confidence intervals are based on 5,000 bootstrap repetitions; the relative effect is the proportion of the total effect accounted for by the corresponding effect.

## 5 Discussion

### 5.1 Analysis of differences in demographic variables

This study found that gender and academic year both exhibited varying degrees of differences in college students’ social support, peer relationships, exercise self-efficacy, and physical activity levels, reflecting the distinct characteristics of different groups in terms of psychological resources and behavioral patterns.

Regarding gender, male college students scored significantly higher than female college students on all variables, with the differences being most pronounced in exercise self-efficacy and physical activity. This result is closely related to long-standing gender role perceptions in China. Influenced by traditional notions, men are often more encouraged to participate in outdoor activities and social interactions, forming broader social networks and becoming more adept at identifying, obtaining, and utilizing external support resources, which in turn translates into greater confidence in exercise and behavioral engagement. In contrast, women may be more reserved in expressing their needs and actively seeking support, which affects their perception of and ability to utilize social support, and may consequently limit the development of exercise self-efficacy and participation in physical activity (Bao, 2025). This difference suggests that gender is not merely a biological classification variable but also an important psychosocial factor influencing health behaviors within a sociocultural framework.

In terms of academic year, first-year students scored significantly higher than second-year students in social support, peer relationships, and the development of self-efficacy, reflecting a “second-year slump”; however, there were no significant differences in physical activity levels across academic years. From a developmental perspective, first-year students are in the early stages of campus adaptation and self-identity formation, exhibiting a strong need for peer connections and external validation, resulting in higher levels of perceived support. In contrast, second-year students gradually enter a phase of academic deepening and career exploration, where their need for support shifts from “broad acquisition” to “precise selection.” This transition, coupled with a natural decline in family support and a shift in peer interactions toward academic discussions, may lead to an overall decrease in perceived social support (Schaller, 2005). Although the differences across academic years were significant, the effect size was small (*η*^2^ < 0.04), indicating that while the academic year factor was statistically significant, its actual impact was limited and likely represented temporary fluctuations during developmental transitions.

### 5.2 The relationship between social support and physical activity

Social support is a direct, positive predictor of physical activity levels among university students, with an effect size of 0.103, accounting for 29.38% of the total effect; Hypothesis H1 is thus supported. This result indicates that the level of support perceived by university students is closely associated with their participation in physical activity. Physical activity during university years gradually shifts from being part of the curriculum to being a matter of personal choice; whether students participate and whether they are able to maintain such activity is more closely tied to their social environment. Recognition, encouragement and feedback from family, teachers and peers not only constitute support resources that university students can perceive, but also convey behavioral signals regarding whether physical activity is valued and whether it meets group expectations. Existing meta-analyses have found a consistent positive association between social support and physical activity among university students, with support from friends showing a stronger association with physical activity than support from family (Wang et al., 2024). This suggests that the relationship between social support and physical activity is not merely reflected in an increase in external assistance, but is also related to how individuals interpret the attitudes of those around them toward physical exercise.

According to social cognitive theory, environmental factors may be directly linked to behavior, or they may be indirectly linked through individual cognition and social interaction (Lu et al., 2024). The direct effect of social support remained significant after controlling for two mediating variables, indicating that peer relationships and exercise self-efficacy can explain part of the statistical association, but do not account for the entire relationship between social support and physical activity. Compared with existing research, which has primarily examined the overall correlation between social support and physical activity, this study further demonstrates that there is both a direct association and an indirect association—mediated by relationships and cognitive factors—between social support and physical activity, thereby providing more detailed evidence for understanding the social contextual foundations of physical activity among university students.

### 5.3 The mediating role of peer relationships

Peer relationships play a significant mediating role between social support and physical activity among university students, with an indirect effect of 0.194, accounting for 55.37% of the total effect; this is the largest effect size estimated among the three indirect pathways, and Hypothesis 2 is supported. This suggests that the association between social support and physical activity is largely linked to the peer relationship context in which university students find themselves. Social support reflects an individual’s perception of overall support resources, while peer relationships embody the relational foundation of these support resources in everyday interactions (Holt-Lunstad, 2018). University students’ study, daily life and leisure activities mainly take place in settings where peers are present, such as classrooms, halls of residence and student societies; positive peer relationships facilitate the exchange of information about physical activity, joint participation and behavioral feedback. Therefore, compared with a broader perception of support, peer relationships are more closely linked to the specific social contexts in which physical activity occurs.

Social cognitive theory posits that individuals form judgments about behavior by observing the actions and outcomes of those who share similar characteristics with themselves. As peers exhibit a high degree of similarity in terms of age, identity and life experiences, their physical activity behaviors serve as strong points of reference. Positive peer relationships entail more frequent interaction, more stable emotional bonds and greater reliability of feedback; consequently, individuals are more likely to perceive their peers’ participation in physical activity as an accessible and imitable behavioral choice. At the same time, shared participation and mutual recognition within peer groups are also linked to the formation of physical exercise norms. When physical activity becomes part of a group’s daily interactions, an individual’s choice of physical activity is no longer merely an isolated personal decision, but becomes intertwined with group belonging and the maintenance of relationships. A longitudinal study by Cao found that changes in peer support were significantly correlated with the developmental trajectories of university students’ total physical activity volume, duration and frequency, which corroborates the findings of this study (Cao et al., 2023).

From the perspective of effect sizes, the peer relationship pathway was significantly stronger than the exercise self-efficacy pathway, which may reflect the fact that physical activity among university students is characterized by a pronounced relational context. Peer relationships are associated not only with exercise role models and group norms, but also with the extent to which physical activity is integrated into students’ daily social interactions; consequently, the link between such relationships and behavior is more direct than that of general assessments of ability. This finding further suggests that the behavioral significance of social support may not depend entirely on the quantity of support resources, but is closely related to whether such support is embedded within stable peer relationships.

### 5.4 The mediating effect of developing a sense of self-efficacy

Exercise self-efficacy plays a significant mediating role between social support and physical activity among university students, with an indirect effect of 0.030, accounting for 8.64% of the total effect; Hypothesis H3 is thus supported. This suggests that social support is not only related to the interpersonal environment in which university students find themselves, but may also be linked to physical activity through their assessment of their own exercise capabilities. Exercise self-efficacy refers to an individual’s assessment of their ability to commence and maintain exercise in situations such as fatigue, time conflicts and environmental constraints. Existing research indicates that self-efficacy is closely related to an individual’s behavioral choices, effort, and coping with difficulties, and is an important individual factor in social cognitive theory’s explanation of physical activity (Young et al., 2014).

The link between social support and self-efficacy in exercise can be understood in terms of how efficacy beliefs are formed. Encouragement and affirmation from others can only be linked to a stable sense of efficacy when the individual interprets them as evidence of their own ability to exercise. General concern and support do not necessarily translate directly into specific assessments of one’s ability to exercise; by contrast, when supportive information is combined with specific exercise tasks, feedback on performance and the individual’s past experiences, it is more likely to reinforce their assessment of their ability to complete and maintain an exercise program (Warner et al., 2014). Therefore, the relationship between social support and self-efficacy in exercise is not a simple matter of resource input, but involves the individual’s recognition and evaluation of supportive information.

The effect size estimates for the ‘exercise self-efficacy’ pathway were lower than those for the ‘peer relationships’ pathway, which may be related to the task-specific nature of self-efficacy. Even when university students perceive a high level of social support, this does not necessarily lead them to form a higher assessment of their exercise ability; conversely, only encouragement, modeling and feedback linked to specific exercise experiences are more likely to be incorporated into an individual’s assessment of their ability. This finding suggests that, in the relationship between social support and physical activity, relational contexts may be closer to the point of behavior occurrence, while the formation of ability beliefs requires more specific cognitive processing. Compared with existing studies that examine the individual effects of social support or self-efficacy in isolation, this study incorporates both into a single model, thereby further illustrating the potential pathways through which external support and individual perceptions of ability jointly influence physical activity.

### 5.5 The chain mediation effect of peer relationships and exercise self-efficacy

Peer relationships and exercise self-efficacy exert a significant chained mediating effect between social support and physical activity among university students, with an indirect effect of 0.023, accounting for 6.61% of the total effect; Hypothesis 4 is thus supported. This suggests that higher levels of social support may be associated with more positive peer relationships, which in turn are associated with higher exercise self-efficacy and are further linked to higher levels of physical activity. Compared with the independent mediation of peer relationships and exercise self-efficacy, the chained pathway reveals not a simple summation of the two mediating variables, but rather a theoretically sequential and continuous association between the social environment, relational contexts and individual cognition.

According to social cognitive theory, environmental factors do not enter an individual’s cognition directly, but are observed, evaluated and interpreted within specific social interactions. Social support is a relatively broad environmental resource, while peer relationships constitute the immediate context in which supportive information is continuously perceived and processed by the individual (McHale et al., 2022). When university students engage in physical exercise alongside peers with whom they have a good relationship, they are more likely to observe the task performance of those with similar physical capabilities and to receive feedback on specific behaviors. The vicarious experience provided by similar peers completing exercise tasks, the sense of progress gained through joint participation, and verbal affirmation from close associates may all serve as sources of information for individuals to assess their own exercise capabilities. Consequently, peer relationships are not only directly related to physical activity but may also form the relational foundation linking social support to self-efficacy in exercise.

The chain mediation pathway further illustrates the cognitive processes underlying the relationship between peer relationships and physical activity, which differ from direct behavioral associations. Peer participation, group norms and invitations to exercise have a relatively direct link with physical activity, corresponding to the independent mediation pathway of peer relationships—which exhibited a stronger effect in this study; modeling, feedback and experiences formed through peer interactions may also be related to an individual’s assessment of their own abilities, and subsequently form a continuous association with physical activity via exercise self-efficacy, corresponding to the chain mediation pathway. The former primarily reflects the embedded relationship between physical activity and the peer context, while the latter further illustrates the link between the peer context and an individual’s perception of their own capabilities. This distinction suggests that peer relationships serve not only as the social context in which physical activity takes place, but may also act as an intermediary link connecting external support with an individual’s perception of their own efficacy.

The chain path accounted for the smallest proportion of the effect among the three indirect paths. This finding suggests that the sequential associations between social support, peer relationships, exercise self-efficacy and physical activity are subject to more specific conditions. Not all social support manifests as improved peer relationships, nor do all peer interactions involve exercise-related modeling, feedback and affirmation of competence. By situating social support, peer relationships and exercise self-efficacy within the ‘environment–individual–behavior’ framework of social cognitive theory, this study reveals how broader social support forms a continuous association with physical activity among university students via proximate peer relationships and task-specific assessments of competence. While previous research has largely examined the independent associations between social support and exercise self-efficacy, or between peer relationships and physical activity, this study further reveals that peer relationships and exercise self-efficacy are not mutually exclusive, but may together constitute a sequential mechanism linking the external social environment to physical activity.

### 5.6 Practical implications

The findings of this study indicate that university efforts to promote physical activity among students should be grounded in social support, with peer relationships serving as the primary point of intervention, and that the development of exercise self-efficacy should be regarded as a key criterion for assessing whether an intervention yields lasting effects. Firstly, universities may establish ‘one-to-one exercise partners’ or small exercise support groups of three to five members, based on students’ sporting interests, free time, physical activity background and skill levels. By setting fixed activity times, shared goals, participation reminders and periodic feedback, general campus support can be transformed into stable peer interactions. Each faculty may select and train student sports leaders to act as peer facilitators; however, their responsibilities should not be limited to demonstrating sporting skills, but should also include actively inviting students with low activity levels, coordinating activity arrangements, providing positive feedback, and fostering a non-judgmental atmosphere for participation.

Secondly, peer activities should incorporate mechanisms for fostering a sense of self-efficacy. Universities can break down exercise goals into achievable, progressively more challenging sub-tasks, allowing students to choose the type of activity and exercise intensity independently based on their physical fitness levels; through exercise logs, stage-based achievement targets and feedback on progress, students can continuously accumulate experiences of mastery; Peers of similar ability levels who demonstrate clear progress should be selected to serve as role models, rather than relying solely on high-level athletes; peer feedback should be guided to focus on effort, strategies and individual progress, minimizing comparisons centered on physical fitness, appearance and competitive results. For female students, those with low activity levels or those lacking confidence in their athletic abilities, introductory courses, female-friendly activity sessions and low-competitive sports should also be provided to lower the psychological barrier to joining peer exercise groups.

Finally, higher education institutions should establish a support network involving physical education teachers, student counselors, student societies and support services, to provide stable conditions for peer-led exercise in terms of access to facilities, booking systems, curriculum integration and the dissemination of activity information. The evaluation of interventions should not merely examine the frequency of activities or short-term physical activity levels; it should also measure the quality of peer interaction, self-efficacy regarding exercise and the sustainability of behavior, and validate these findings using wearable devices or exercise logs. By comparing different intervention strategies—such as ‘providing exercise resources only’, ‘increasing peer support’ and ‘peer support combined with self-efficacy training’—it is possible to further determine the actual contribution of each component, thereby establishing a model for promoting physical activity in higher education institutions centered on the sequence of ‘environmental support – peer connections – self-efficacy enhancement – behavior maintenance’.

## 6 Conclusion

This study found that: (1) There were significant positive correlations between all pairs of the four variables—social support, peer relationships, exercise self-efficacy and physical activity among university students. (2) Social support significantly and positively predicted the level of physical activity among university students and is an important external resource for promoting their physical exercise behavior. (3) Peer relationships and exercise self-efficacy each played a simple, independent mediating role between social support and physical activity. (4) Peer relationships and exercise self-efficacy also constitute a chained mediation pathway between social support and physical activity; that is, social support promotes physical activity participation among university students by optimizing peer relationships, which in turn enhances exercise self-efficacy.

### 6.1 Limitations and future directions

This study has the following limitations. Firstly, cross-sectional data cannot determine the temporal sequence or direction of causality between social support, peer relationships, exercise self-efficacy and physical activity. Although this study controlled for gender and employed HC3 robust standard errors, Bootstrap confidence intervals and non-linear sensitivity analyses, the influence of unmeasured confounding factors cannot be entirely ruled out; the results of the chained mediation analysis should be interpreted as indirect statistical associations between variables rather than causal mechanisms. Secondly, as all variables were measured using self-administered questionnaires at a single point in time, there may be recall bias, social desirability bias and common-method bias; the Harman one-factor test cannot entirely rule out these issues. Although the PARS-3 covers exercise intensity, duration and frequency, its internal consistency is at the lower limit of acceptable standards (Cronbach’s *α* = 0.705), and it does not distinguish between activity domains such as work, commuting, housework and leisure. Consequently, the study results can only reflect the overall volume of physical exercise among university students and cannot be generalized to encompass all daily physical activity. Finally, as the sample was drawn solely from five higher education institutions in Province A, and information regarding institution type, academic specialization, urban or rural background, and socio-economic status was not systematically collected, caution should be exercised when generalizing the study’s findings. Furthermore, this study examined only the overall level of social support and two mediating pathways—peer relationships and exercise self-efficacy—and therefore does not yet encompass the full range of associations between social support and physical activity. Future research could adopt a cross-regional longitudinal design, combining objective measures of physical activity with multi-source data, and further distinguish between the sources and dimensions of social support.

## Data Availability

The raw data supporting the conclusions of this article will be made available by the authors, without undue reservation.
